# Shaping the Future of Mental Health Care with Artificial Intelligence: A Narrative Review

**DOI:** 10.1192/j.eurpsy.2026.11359

**Published:** 2026-07-31

**Authors:** M. S. Cunha, C. Batista, G. Lacerda, T. Cardoso, A. F. Reis, Â. Ferreira, J. Miranda, J. Marta, J. Nogueira

**Affiliations:** Arrábida’s Local Health Unit, Setúbal, Portugal

## Abstract

**Introduction:**

The rapid development of Artificial Intelligence (AI) has revolutionized multiple sectors, particularly in medicine. In the field of Mental Health (MH), AI has improved diagnostic accuracy and opened new paths for personalized medicine. At present, MH care faces a crisis driven by increasing demand and limited resources. AI technologies provide predictive analytics, therapeutic tools, clinical decision support, and monitoring systems. Their integration is reshaping how mental disorders (MDs) are diagnosed, treated, and managed, while raising ethical, privacy, and transparency concerns.

**Objectives:**

To review recent advances in AI applied to MH, focusing on diagnostic, therapeutic, and monitoring applications, and to discuss their benefits, limitations, and ethical implications.

**Methods:**

Narrative review of the literature using electronic databases such as PubMed and Scopus, with the keywords “Artificial Intelligence” AND “Mental Health.”

**Results:**

Studies indicate that AI offers multiple clinically useful applications through conversational agents (CAs), machine learning algorithms and digital phenotyping (DP), which support diagnosis and treatment of MDs. CAs, powered by natural language processing, provide personalized interventions, monitor mood, and enhance treatment adherence. Certain AI algorithms are used to predict treatment outcomes and support exposure therapy for phobias (e.g., AI combined with virtual reality). DP enables passive data collection from devices (e.g., GPS, heart rate), which aids in monitoring behaviors and early warning signs. However, most of these applications remain in experimental phases.

**Conclusions:**

AI holds potential to enhance early detection, treatment personalization, and resource allocation in MH care. However, current evidence is preliminary and requires validation in larger, long-term studies. Responsible integration must prioritize transparency, fairness, and data protection, involving MH professionals, patients, and developers in the co-creation of tools that strengthen humanized care. AI should be regarded as a complement to clinical judgment, contributing to a more effective, accessible, and person-centered mental health model.

**Disclosure of Interest:**

None Declared

